# Sensorimotor adaptation of locomotor synergies to gravitational constraint

**DOI:** 10.1038/s41526-024-00350-2

**Published:** 2024-01-11

**Authors:** Etienne Guillaud, Vincent Leconte, Emilie Doat, Dominique Guehl, Jean-René Cazalets

**Affiliations:** 1 Univ. Bordeaux, CNRS, INCIA, UMR 5287, F-33000 Bordeaux, France; 2 Univ. Bordeaux, CNRS, IMN, UMR 5293, F-33000 Bordeaux, France

**Keywords:** Neuroscience, Aerospace engineering, Human behaviour

## Abstract

This study investigates the impact of gravity on lower limb muscle coordination during pedaling. It explores how pedaling behaviors, kinematics, and muscle activation patterns dynamically adapts to changes in gravity and resistance levels. The experiment was conducted in parabolic flights, simulating microgravity, hypergravity (1.8 g), and normogravity conditions. Participants pedaled on an ergometer with varying resistances. The goal was to identify potential changes in muscle synergies and activation strategies under different gravitational contexts. Results indicate that pedaling cadence adjusted naturally in response to both gravity and resistance changes. Cadence increased with higher gravity and decreased with higher resistance levels. Muscular activities were characterized by two synergies representing pull and push phases of pedaling. The timing of synergy activation was influenced by gravity, with a delay in activation observed in microgravity compared to other conditions. Despite these changes, the velocity profile of pedaling remained stable across gravity conditions. The findings strongly suggest that the CNS dynamically manages the shift in body weight by finely tuning muscular coordination, thereby ensuring the maintenance of a stable motor output. Furthermore, electromyography analysis suggest that neuromuscular discharge frequencies were not affected by gravity changes. This implies that the types of muscle fibers recruited during exercise in modified gravity are similar to those used in normogravity. This research has contributed to a better understanding of how the human locomotor system responds to varying gravitational conditions, shedding light on the potential mechanisms underlying astronauts’ gait changes upon returning from space missions.

## Introduction

Gravity is one of the main constraints that applies to terrestrial locomotion. This challenging task requires moving at various speeds while reducing the energy cost of the movement as much as possible^1^. While the risk of falling makes it essential to take gravity into account during bipedal walking, this is less true during a pedaling movement thanks to the maintenance of the body by external supports other than the pedals (saddle, handlebars, etc.) which allow stabilization. Both walking and pedaling results from the rhythmic coordinated and highly reproducible muscular activities that are centrally generated and many similarities have been put forward between the muscle activity patterns of walking and pedaling^2,3^.

Muscle coordination during pedaling has often been described in terms of synergies to provide a simplified view of motor patterns by reducing the dimensionality of motor behaviors^4–6^. This type of analysis supports the hypothesis that the central nervous system (CNS) generates a reduced number of patterns, as several muscles can be activated synchronously during the same phase of a cycle. Each synergy is described by (1) its activation coefficient which represents the relative contribution of the synergy to the overall muscle activity during one cycle (i.e., during a pedal revolution) ; (2) a muscle vector that specifies the relative weight of each muscle corresponding to these activation coefficients. In a study based on electromyographic analysis of 11 leg muscles during pedaling, Hug et al. ^7^. showed that the structure of these synergies (coefficients and vectors) was little modified by the force required, the pedaling speed or the posture of the participants. This observation held true even when analyzing both legs^8^. A recent study by Cartier et al. ^9^. showed that upper limb pedaling exhibited a greater diversity of muscle patterns than lower limb pedaling, with greater inter-individual variability in the synergies identified. One of the hypotheses put forward to explain this difference is that the control of the lower limbs is optimized for its antigravity role, which would favor the coordination of a downward thrust on the pedal, whereas the upper limbs would be involved in a greater diversity of movements (reaching, grasping, manipulating…). Very few studies have, however, investigated the impact of gravity on muscle synergies.

It has been shown that the CNS takes the gravity context into consideration during motor programming of ballistic movements, in visuo-manual pointing^10^, to maintain intersegmental kinematics^11^ as well as to minimize efforts during movement production^12^. However, only subtle changes were observed in the synergy that would underlie the postural adjustments preceding arm movement^13^, and no change in synergies involving upper limb reaching movement was revealed. Similarly, during postural perturbations in partial gravity or hypergravity, only minor changes were observed in synergies compared to normogravitational postural context^14^. In contrast, for upper-limb cyclic movements on independent cranks, major changes in synergies were observed when the body position was changed (supine vs. sitting position), suggesting an impact of the gravitational vector orientation on muscular coordination^15^. As regards to locomotor movements, the impact of gravity on muscle coordination was never reported. Only a simulated Martian gravity experiment^16^ showed that a walking pattern was not modified by hypo-gravity, and predictive numerical simulations^17^ suggest that skipping is more efficient and less fatiguing than walking at low gravity. A gait experiment conducted under harness^18^ unveiled that synergies orchestrating muscles activation patterns are consistent across various body weight compensations. While partial changes were noted, indicating distinct impacts on synergies acting during the supporting phase compared to those preceding foot contact, the approach had its limitations. Despite a reduction in biomechanical demand during the stance phase, all body segments remained subject to gravity constraints during their displacements, and sensorial feedback accounted for normogravity. An other experiment from NASA^19^ on the enhanced zero-gravity locomotion stimulator report only minor difference in locomotion patterns and muscular activities between different partial loading mechanism, without available comparison to normo-gravity pattern.

When astronauts return from space missions, their gait is nevertheless modified^20,21^ but many factors can explain this difference in behavior (muscle loss, progressive readaptation to gravity, etc.) and an adaptation of the muscle coordination pattern during space flight has not been highlighted. After a space mission, a decrease in neuromuscular action potential discharge frequencies has been shown with, however, a force production identical to pre-mission tests. This effect is similar to that of a period of bedrest^22^ or confinement^23^. This decrease in median electromyogram frequency may be related to a change in the muscle typology^24^ of the spacewalkers, as is also observed when muscle activity is permanently reduced (^25^ for a review). However, astronauts maintain significant physical activity, e.g. 2.5 h per day during missions on the International Space Station, including the time spent using the CEVIS cycloergometer^26^. This raises the question of a change in the typology of muscle fibers used during pedaling in microgravity, which could explain these changes despite regular training. As energy constraints and expenditure are modified, it is possible that the muscle fibers solicited are not the same in different gravity contexts. A favored use of type Ia muscle fibers (slow, oxidative metabolism, activated by moderately sized motor neurons with slow conduction speed) in microgravity, to the detriment of the use of fast type II fibers (motor neurons discharging at higher frequency) could explain this decrease in the median frequency of EMG.

**Fig. 1:**
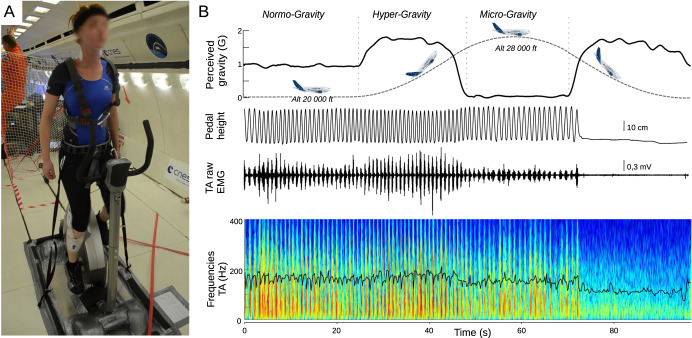
Experimental setup. **A** Participant performing the pedaling task, in an erect position, arms at sides, held vertically at hip level by straps. **B** Example of recorded signal during a parabola. The participant pedaled in normogravity, then in the first hypergravity phase and in microgravity. Perceived acceleration, pedal position and EMGs were measured. The bottom panel shows the frequency analysis of the EMG signal presented just above (TA); the black curve corresponds to the instantaneous median frequency used in the iMNF calculation.

So far, the coordination patterns of locomotor activities without gravity constraints have never been documented. These rhythmic activities are generated by innate spinal generators^27^, and are permanently adapted to the different constraints encountered in terrestrial life^28,29^. However, is the “free” expression of the spinal generators, i.e., without any enforced cadence or constraint, different from that encountered on earth? Does our locomotor system immediately adapt to a hyper-gravity or a total absence of gravity ? If so, is the pedaling cadence modulated and are the muscular coordination patterns of the locomotor system modified? The objective of the present study was therefore, to identify the impact of a gravity change on lower limb muscle coordination during a pedaling task. For this purpose, electromyographic recordings were performed in participants during parabolic flights in normo-, hyper- (1.8 g) and microgravity (0 g). Muscle synergies were extracted using non-negative matrix factorization^6^. Despite the expected robustness of the locomotor synergies studied, we hypothesized that gravity modulation would influence the participants’ preferential pedaling rhythm, as well as the temporal coordination of the propulsor (pressing down on the pedal) and lifter (pulling up the opposite leg) muscle groups. We also hypothesized that the frequencies of neuromuscular discharges observed in electromyography would be reduced in microgravity Fig. 1.

## Results

### Pedaling cadencies

**Fig. 2:**
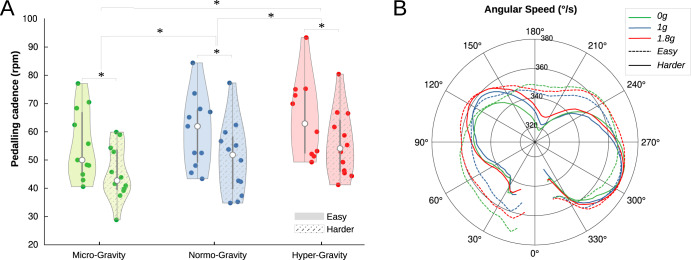
Pedaling kinematic. **A**The cadence was increased when gravity increased, and was decreased when the resistance was harder. **B** Angular pedaling speed over the cycle (to help the visual comparison of kinematic profile, the velocity from all gravitational conditions were normalized to have the same average). Speed variations over the cycle were pronounced when resistance was increased and less so when resistance was reduced, but these kinematic profiles were little affected by changes in gravity. The missing part of the curve (around 0) corresponds to the potentiometer’s blind zone.

Both Gravity and Resistance significantly affected the pedalling cadencies, without interaction between the two factors (F(2,20) = 0.31; *p* = 0.73; Fig. 2A). Cadence decreased when the pedalling resistance increased, from 60.2 rpm (SD 13) to 50.5 rpm (SD 11.2) in the *Easy* and *Harder* conditions respectively (F(1,10) = 41.9; *p* < 0.001). When the gravity increased, the cadence increased, with values of 50.3 rpm (SD 10.9) in microgravity, 55.4 rpm (SD 12.8) in normo-gravity and 60.4 rpm (SD 12.6) in hypergravity (F(2,20) = 25.7; *p* < 0.001). Comparison of pedaling cadences during the first parabolas (first 10 cycles of each condition) did not reveal a main effect of primacy (F(1,10) = 2.15; *p* = 0.17), and there was no observed interaction with gravity (F(2,20) = 2.94; *p* = 0.08) or resistance (F(1,10) = 0.35; *p* = 0.57).

### Angular velocity

Inside cycles, angular velocity varied, with higher velocity observed during the propulsive phase (Fig. 2B). Regarding the effects on cadencies, the cycle velocities were significantly affected by gravity (averaging both resistance conditions), with minimal and maximal velocities ranging from 273°/s (SD 84) to 307°/s (SD 78) in 0 G, 301°/s (SD 87) to 334°/s (SD 87) in 1 G, and 332°/s (SD 85) to 370°/s (SD 89) in 1.8 G (F(2,18) = 15.8, *p* < 0.001 for minimal velocities ; F(2,18) = 22.8, *p* < 0.001 for maximal velocities). Minimal and maximal cycle velocities were also significantly affected by Resistance (all conditions averaged), ranging from 346°/s (SD 79) to 369°/s (SD 85) in *Easy* condition, and 268°/s (SD 79) to 310°/s (SD 82) in *Harder* condition. The velocity variation ratio was significantly higher when the resistance increased, with values of 8.9% (SD 3.5) in *Easy* condition and 15.7% (SD 7.2) in *Harder* condition (F(1,8 = 7.5, *p* = 0.02). This ratio was not affected by gravity, with values of 13.7% (SD 9.8), 12.4% (SD 5.6) and 12% (SD 3.7) in G0, G1 and G2 conditions, respectively (F(2, 16) = 0.12, *p* = 0.88). No interaction between gravity and resistance was measured on velocity ratio (F(2, 16) = 1.56, *p* = 0.24). The angular position corresponding to the velocity peak was not affected by gravity (F(2, 18) = 2.15, *p* = 0.14) or resistance (F(1, 9) = 1.7, *p* = 0.22), and was 283° (4 o’clock, SD 40) in average. The velocity ratio extracted from the first 10 cycles was not found to be significantly different from the subsequent cycles (F(1,10) = 0.65; *p* = 0.47), and no interaction with gravity (F(2,20) = 0.94; *p* = 0.41) or resistance was observed (F(1,10) = 0.06; *p* = 0.81).

### EMG Amplitude

**Fig. 3:**
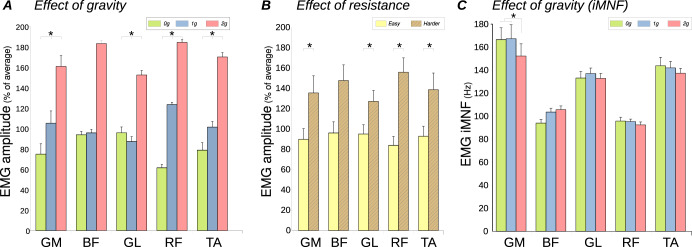
Electromyographic datas from all five muscles. (GM gluteus maximus; BF biceps femoris; GL lateral gastrocnemius; RF ectus femoris; TA tibialis anterior). Error bars represent standard error. **A** Main effect of gravity on EMG amplitude (amplitude increased when gravity increased). **B** Main effect of resistance on EMG amplitude (amplitude increased when resistance increased). **C** Instantaneous Median Frequency (iMNF). IMNF was not affected by resistance (not illustrated), and a marginal effect of hypergravity was observed on GM only.

In all five muscles, the EMG amplitude increased when gravity increased (see Fig. 3A). This main effect of Gravity was significant for GM (F(2, 20) = 3.7, *p* = 0.04), GL (F(2, 20) = 3.7, *p* = 0.04), RF (F(2, 8) = 8.9, *p* = 0.009) and TA (F(2, 20) = 7.7, *p* = 0.003), but not for BF (F(2, 8) = 1.6, *p* = 0.25). An effect of Resistance (Fig. 3B) was also observed, with increased amplitude in *Harder* compared to *Easy*. This main effect of Resistance was also significant for GM (F(1, 10) = 5.3, *p* = 0.04), GL (F(1, 10) = 6.7, *p* = 0.027), RF (F(1, 4) = 17, *p* = 0.015) and TA (F(1, 10) = 3.4, *p* = 0.003), but not for BF (F(1, 4) = 3.89, *p* = 0.12). For all five muscles, no interaction was observed between gravity and resistance (GM : F(2,20) = 0.1, *p* = 0.89; BF : F(2,8) = 2.5, *p* = 0.14; GL : F(2,20) = 3.06, *p* = 0.07; RF : F(2,8) = 1.55, *p* = 0.27; TA : F(2,20) = 0.26, *p* = 0.78). Comparison of EMG amplitudes from the last 10 cycles with the first 10 cycles of each condition did not reveal a main effect period (GM : F(1,10) = 0.11, *p* = 0.75; BF : F(1,4) = 1.7, *p* = 0.26; GL : F(1,10) = 0.64, *p* = 0.44; RF : F(1,4) = 0.001, *p* = 0.97; TA : F(1,10) = 2.2*, p* = 0.17), and there was no observed interaction with gravity or resistance for all five muscles.

### EMG frequency

We observed a significant decrease in the median of instantaneous frequencies (iMNF) when gravity increased (Fig. 3C) for GM (F(2,20) = 5.3, *p* = 0.014). The iMNF values were 167 Hz, 167 Hz and 152 Hz for 0 G, 1 G and 1.8 G respectively, with no significant difference between 0 G and 1 G. A main effect of gravity was also measured on iMNF for BF (F(2,8) = 4.7, *p* = 0.044, with iMNF values of 94 Hz, 104 Hz, and 106 Hz for 0 G, 1 G, and 1.8 G, respectively. However, post hoc did not reveal any significant difference between all 3 gravity conditions for BF. For both GM and BF, we did not observe any significant effect of resistance on iMNF (GM: F(1, 10) = 0.66, *p* = 0.44; BF: F(1, 4) = 2.8, *p* = 0.17). Additionally, there was no significant interaction between gravity and resistance for both muscles (GM: F(2, 20) = 1.13, *p* = 0.34; BF: F(2, 8) = 0.17, *p* = 0.85). For GL, RF and TA, neither the effect of gravity (GL : F(2,20) = 0.85, *p* = 0.44; RF : F(2,8) = 1.76, *p* = 0.23; TA : F(2,20) = 1.31, *p* = 0.29) nor the effect of resistance (GL : F(1,10) = 0.08, *p* = 0.78; RF : F(1,4) = 0.004, *p* = 0.95; TA : F(1,10) = 0.14, *p* = 0.72), nor the interaction (GL : F(2,20) = 0.56, *p* = 0.58; RF : F(2,8) = 0.013, *p* = 0.98; TA : F(2,20) = 1.36, *p* = 0.28) was observed. The frequencies were 134, 94 and 131 for GL, RF and TA respectively. For all five muscles, the iMNF from the last 10 cycles of each condition did not differ from the initial 10 cycles (GM : F(1,10) = 1.88*, p* = 0.2; BF : F(1,4) = 1.0, *p* = 0.37; GL : F(1,10) = 2.87, *p* = 0.12; RF : F(1,4) = 0.04, *p* = 0.84; TA : F(1,10) = 1.35, *p* = 0.27), and there was no observed interaction with gravity or resistance.

### Muscular synergies

**Fig. 4:**
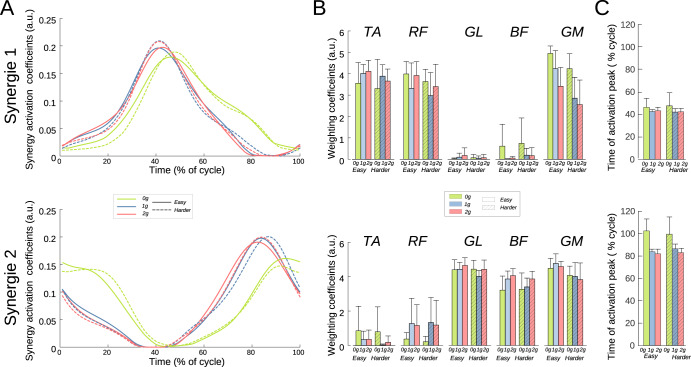
Synergies extracted and averaged from all the participants. Two synergies were enough to explain the activities of the five muscles under all gravitational and resistive conditions. Synergy 1 corresponded to the pull phase and synergy 2 to the push phase. Error bars represent standard deviation. **A** Activation coefficients (in arbitrary unit) during one cycle (pedal revolution). Coefficients were affected by microgravity (green curves). **B** Weighting coefficients (in arbitrary unit), that illustrate the contribution of the synergy to the activity of the considered muscle. TA and RF were mainly involved in synergy 1 (pull), while GL and BF were mainly involved in synergy 2 (push). **C** Time of activation peak, that correspond to the time of occurrence of the higher peak of activation coefficient in the considered synergy. In both synergies, this appearance was delayed for microgravity compared with the other two gravitational conditions.

On the five recorded muscles, two synergies were found to be adequate in explaining more than 90% of the variance across the different conditions. The first synergy (see Fig. 4) corresponds to the pull phase of the leg, with activation coefficients that were maximal at 43% of the cycle on average just before reaching the top position of the foot (160°, 11 o’clock). Conversely, the second synergy corresponds to the push phase, with a distinct activation peak occurring at approximately 90% of the cycle (328°, 4 o’clock), just before the down position.

In synergy 1, the muscles involved were the TA, RF and RM muscles. The GL and BF muscles exhibited negligible weighting coefficient under all conditions (Fig. 4B upper panel). The weighting coefficients of TA and RF were unaffected by resistance (TA : X^2^ (1, 24) = 0.29, *p* = 0.59; RF : X^2^ (1, 24) = 3, *p* = 0.08), and gravity (TA : X^2^ (2, 24) = 1.12, *p* = 0.57; RF : X^2^ (2, 24) = 2.08, *p* = 0.35) with average values of 3.76 and 3.53 a.u. for TA and RF, respectively.

For GM, the weighting coefficients of synergy 1 significantly decreased as gravity increased (X^2^ (2, 24) = 9.88, *p* = 0.007). The values were as follows: g0 = 4.6 a.u., g1 = 3.5 a.u., g2 = 3 a.u. (all differences were significants). Moreover, resistance had a significant effected on the coefficients attributed to GM in synergy 1 (X^2^ (1, 24) = 6.11, *p* = 0.013), with higher values observed in the *Easy* condition compared to the *Harder condition* (4.2 a.u. vs. 3.2 a.u.).The activations coefficients of synergy 1 were influenced by gravity but not by resistance. The peak of the activation coefficient (Fig. 4C) occurred later in g0, compared to g1 and g2 (47%, 43% and 43% respectively; X^2^ (2, 24) = 8.4, *p* = 0.014, with no significant difference between g1 and g2). Resistance did not modified these occurrence (X^2^ (1, 24) = 0.03, *p* = 0.85). The peak magnitude was not significantly affected either by gravity (X^2^ (2, 24) = 3.1, *p* = 0.21), or by resistance (X^2^ (1, 24) = 3.5, *p* = 0.06; grand average = 0.2).

Synergy 2, corresponding to the push phase, primarily modulated the activity of GL, BF and GM, while TA and RF were minimally affected (Fig. 4B lower panel). Weighting coefficents of GL and GM were not significantly modified by either resistance (GL : X^2^ (1, 24) < 2.27, *p* = 0.13; GM : X^2^ (1, 24) = 2.27, *p* = 0.13), or gravity (GL : X^2^ (2, 24) = 5.07, *p* = 0.08; GM : X^2^ (2, 24) = 0.36, *p* = 0.84) with average values of 4.4 a.u. and 4.3 a.u. for GL and GM, respectively. The weighting coefficient of BF in synergy 2 was not significantly modulated by resistance (X^2^ (1, 24) < 0.1.6, *p* = 0.21), but it significantly increased when gravity increased (X^2^ (2, 24) = 6.1, *p* = 0.048; with values of 3.2, 3.6 and 4 for g0, g1 and g2 respectively).

Similar to synergy 1, activation coefficients of synergy 2 were only influenced by gravity. The peak of the activation coefficient (Fig. 4C lower panel) occurred later in g0, compared to g1 and g2 (101%, 85% and 83% respectively, X^2^ (2, 24) = 15.3, *p* < 0.001, all differences significant). Resistance did not significantly modify these occurrence (X^2^ (1, 24) = 0.45, *p* = 0.5). Likewise, peak magnitude was significantly affected by gravity (X^2^ (2, 24) = 7.1, *p* = 0.03; with a lower peak in g0 compared to g1 and g2, with values of 0.18, 0.21 and 0.2, respectively), but not by resistance (X^2^ (1, 24) = 1.6, *p* = 0.2).

## Discussion

The comparison of muscular activities and pedaling kinematics in a free pedaling task under various gravitational conditions has not been previously documented. This study aimed to observe participants’ natural behaviors during repeated pedaling sequences under normogravity, hypergravity, and microgravity conditions, incorporating both resistance-free and medium-resistance pedaling sequences. The results indicated that gravity and pedaling resistance were two distinct factors that significantly influenced pedaling behavior, albeit in separate and independent manners.

The initial striking observation is the natural adjustment of cadence in response to changes in both resistance and gravity. These effects remained consistent throughout the entire experiment, and we did not observe any kinematic differences between the first pedaling cycles and the subsequent ones in the experiment. Cyclists commonly decrease their cadence as resistance increases, a phenomenon well-known among cyclists. During uphill cycling, riders intuitively enhance power output while reducing cadence, aiming to achieve the optimal cadence that matches the required external power^30,31^. Notably, the effect of gravity on pedalling cadence variations has never been reported before. In our measurements, we observed an increase in cadence with an increase in gravity. This trend was evident not only in a deceleration during microgravity but also in an acceleration under hypergravity conditions, independent of the resistance level. Consistent findings were reported by White et al. ^32^. on cyclic arm movement (one-handed drawing of the infinity symbol). In their study, the effect of gravity on movement cadency were closely associated with the resonance frequency of the system. This frequency is adopted by the central pattern generator (CPG) within contextual constraints, optimizing energy exchange with the environment. In the absence of gravitational constraints, such as in weightlessness, a diminished impact of the resonant frequency allowed for a more uninhibited expression of the CPG’s innate rhythm. Our focus was on observing the unconstrained expression of the locomotor CPG, and our results indicate a spontaneous decrease in cadence when gravity was absent. This trend held true for both the kinematics and coordinated muscular activities, suggesting that the locomotor coordination is hurried by gravitational constraint, both on Earth and in hypergravity. Interestingly, the observed cadence increase in hypergravity was associated with a heightened sense of effort perceived by the participants. This contrasts the pattern observed with resistance changes where increased resistance led to both heightened perceived difficulty and decreased cadence. Moreover, the cadence increase associated with higher gravity was accompanied by a rise in muscular activity, as indicated by increased EMG amplitudes. This also contrasts with the response to increased resistance, where muscle activity increases while cadence decreases. The interplay between muscular activity and cadence, which changes in tandem with gravity shifts but diverges with resistance changes, suggests that the effects of gravity are distinct from those produced by an increase in pedaling power. This differentiation is also evident in pedaling kinematics. While pedaling speeds vary across conditions, a deceleration phase is consistently observed when the right foot is in the upper position, followed by an acceleration phase culminating in maximum speed between the front and bottom positions. These two phases are more pronounced under higher resistance compared to lower resistance, regardless of the prevailing gravity, as underscored by the significant impact of resistance on the velocity variation ratio. Notably, the velocity profile remains unaffected by changes in gravity, emphasizing the distinction between changes in gravity and changes in effort.

The observed stability in the velocity profile across different gravitational conditions, despite variations in cadence, is of particular interest when linked to the locomotor activation pattern. The decomposition of muscular activities has revealed two distinct synergies, one corresponding to the pull phase and the other to the push phase. Notably, the lower velocity period (occurring just after the foot is lifted, at 55% of the pedal cycle) aligns with the transition of activity between the pull and push synergies. Similarly, the period of maximal velocity (front-bottom foot, 55% of the cycle) coincides with the peak activity of the push synergy. While velocity profiles remain stable, the activation coefficients of both pull and push synergies were significantly impacted by changes in gravity. Specifically, in both synergies, the activation coefficients were delayed by 5 to 15% of the cycle in microgravity compared to normo- and hyper-gravity conditions. Importantly, this delay was unique to microgravity, as no differences were observed in activation coefficients between normo- and hyper-gravity. These observations suggest that the central nervous system (CNS) spontaneously prioritizes the stability of kinematic output and adjusts the muscular pattern accordingly. This adaptation in microgravity is characterized by a delay in muscle activation relative to the foot’s position. It is worth noting that the delayed activation coefficients in microgravity differ from the effects observed on cadence, which exhibited a more linear response to changes in gravity, increasing consistently between micro- and normo-gravity as well as between normo- and hyper-gravity. This indicates that the alterations in synergies observed in microgravity cannot be solely explained by changes in cadence, aligning with observations made on Earth under different mechanical conditions^7,8^. Moreover, similar cadences were achieved despite modified synergies, as exemplified by the *Microgravity-Easy* and *Hypergravity-Harder* conditions. As we observed an influence of gravity on the amplitude of electromyographic (EMG) signals, it raises the question of how power requirements may impact synergies. Earlier studies suggested that alterations in power requirements do not influence lower cycling synergies^7,8^. Our findings align with these prior observations, as we noted comparable EMG amplitudes supported by slightly different synergies in *Microgravity-Harder* and *Normogravity-Easy* conditions. In contrast, similar synergies could support unequal EMG amplitudes in *Normogravity-Harder* and *Hypergravity-Easy* conditions.

Changes in kinematic profiles, pedaling cadence, and EMG amplitude do not appear to be the underlying factors driving the observed changes in synergies in microgravity. It seems that the CNS adapts the synergies to ensure a stable motor output across various gravitational conditions. One of the primary effects of gravity on human movement control is the alteration of body weight. In the context of cycling, the seated position eliminates the risk to balance, as participants are seated securely. Both legs undergo alternating upward and downward movements, and while gravity facilitates the descent of one foot, it simultaneously hinders the ascent of the opposite leg. Given the mechanical linkage between the pedals, the potential advantages and disadvantages of gravity-induced force modifications potentially offset each other. While the forces generated to push a pedal with one leg might aid in pulling the opposite side, the CNS must deal with this constraint to determine the optimal activation pattern. On Earth, when a pedal is pushed downward, the weight of the pushing leg assists in generating force, and the weight of the upper body acts as a fulcrum. This is especially evident during uphill cycling, where the increased power demands encourages cyclists to pedal in a dancer style, a way of loading the downward pedal with all their weight. In microgravity, however, the positions of segments and body weight distribution have minimal impact on pedal pressure, the parameters were changed for the CNS. The optimal muscular pattern required to achieve the desired power output may change, potentially explaining the observed differences in synergies. One can note that the variation of velocity during a pedal revolution remain the same in micro-gravity, which suggests that this velocity profile is not solely a result of the leg and upper body weight assisting in pedal descent. In contrast, changes in angular velocity were influenced by resistance, indicating that they stem from effector configuration (joint angles and muscular stretch), rather than the utilization of body weight. In summary, the velocity profile and muscular activation intensities are tailored to meet the required power output. When gravity changes, the CNS adjusts muscular coordination to maintain stable output, despite changes in body weight and fulcrum. However, for a more comprehensive understanding of the role of whole body weight in power production during cycling, further investigation of upper body kinematics and muscular activity is necessary.

The investigation into muscular fiber usage during exercises performed in microgravity holds significant importance, particularly due to astronauts’ reported difficulties in walking upon returning to Earth, even after engaging in regular and intense locomotor activity during space missions. Electromyography (EMG) serves as a non-invasive means of assessing muscular fiber typology, and thus, we computed instantaneous median frequency (iMNF) from our participants. The aim was to identify potential shifts in muscular fiber type utilization during exercise training in microgravity, which could potentially underlie the observed changes in astronauts’ conditions following space missions. Parabolic flights allowed us to conduct repeated measurements under (artificially) modified gravity conditions, with each of the three gravitational contexts repeated every three minutes. This design minimized the potential fatigue effects when transitioning between different gravitational conditions. Notably, between the first and last parabolas, we did not observe electromyographic fatigue markers, characterized by a decrease in iMNF accompanied by an increase in EMG amplitude^33,34^. In this study, the influence of gravity on neuro-muscular discharge frequencies was found to be negligible. This held true from the initiation of the task to its completion. For the two muscles in which iMNF was found to be influenced by gravity (GM and BF), no significant differences were observed between microgravity and normogravity. Thus, based on our results, there is no indication that the fibers utilized during exercise in modified gravity differ from those employed in normogravity, even after a moderate exposure time (5 minutes of microgravity for the entire experiment). However, it’s important to note that participants were exposed to microgravity for only short periods, interspersed with hyper- and normogravity. This intermittent exposure could hinder the neuro-muscular adaptation process, limiting our ability to draw conclusions about the functioning of astronauts who have experienced long-duration weightlessness during extended space missions.

The pedaling task serves as a physical activity employed by astronauts to counteract body mass loss and address changes in body composition^35^. The lower EMG amplitudes recorded in microgravity may indicate a requirement for higher resistance on the ergometer to compensate for the reduced power demand. However, the notable reduction in cadence observed in microgravity, especially with increased resistance, raises the risk of excessively low cadences when both effects are combined. Simultaneously, the observed changes of synergies in microgravity, which resemble a temporal shift between muscular activation and motor output, are unlikely to affect the energetic benefits of cycling. One way to restore cycling activity in microgravity, comparable to that in normal gravity, could involve recreating vertical forces acting on different parts of the body. Wearing weights to increase inertial forces on the thighs and shins, or using elastic straps pulling in the usual gravitational axis, could be potential solutions. However, as mentioned earlier, the key difference may lie in the loss of the support provided by the body weight in the presence of gravity. Restoring a fulcrum at the hip or shoulder level could be the solution. In this regard, it is interesting to note that the support provided to astronauts using the CEVIS has evolved over time, shifting from a belt at the pelvic level to support bars reestablishing support at the shoulder level. This preference suggests that axial activities play a crucial role in pedaling, an aspect that would be interesting to explore by measuring trunk muscle activity during pedaling, whether in microgravity with different points of support or in normal gravity with partial unloading through suspension at various levels (hip, shoulders, arms). However, it remains unlikely that replicating muscular activity perfectly similar to that performed on Earth would be sufficient to prevent the consequences of prolonged exposure to microgravity, given the critical importance of the concept of total energy expenditure.

This study represents the pioneering effort to evaluate locomotor synergies in both microgravity and hypergravity conditions. The observed spontaneous behaviors indicate that an adaptation process occurs in response to the new gravitational environments, which extends beyond a mere adjustment in power requirements. Parameters such as cadence, EMG amplitude, and the characteristics of Push and Pull synergies were all influenced, underscoring the complexity of human motor control during altered gravitational states. Our findings strongly suggest that the CNS dynamically manages the shift in body weight by finely tuning muscular coordination, thereby ensuring the maintenance of a stable motor output.

## Methods

Twelve healthy adults (5 women and 7 men) volunteered to participate in this study (age: 38 ± 7 yr). They provided written informed consent prior to participating in each experiment. The participants had no prior experience in cycling as a sport or on a regular basis. The procedures followed guidelines from the Declaration of Helsinki and were approved by the French National Research Ethics Review Board (Comité de Protection des Personnes Sud-Ouest et Outre Mer III) under agreement number 2011-A00424-37. All the experiment, from recording to statistical outputs, have been completed using Matlab 2021a (The Mathworks, Natick, USA).

### Micro- and hyper-gravity

This experiment occurred in the Zero-G airplane (A300, Novespace, Mérignac, France) chartered by the Centre National d’Etudes Spatiales (CNES, France) during two parabolic flight campaigns (VP-102 and VP-115; 6 flights in total). During each flight, 30 parabolas were performed, and two subjects were tested (15 parabolas per subject). Each parabola provided a 22 seconds zero gravity time window, preceded and followed by 20 seconds of increased gravity (1.8 g; Fisk et al. 1993). The subjects were medicated using scopolamine, which despite its influence on vestibular inputs and motor control^36–38^, probably did not affect the cycling behavior. A tri-axial accelerometer (ID1049, Phidgets, Calgary, Canada) was attached to the aircraft, to precisely identify these different phases in the analysis.

### Pedaling task

In this experiment, participants had to produce 15 sequences of continuous pedaling during 90 s, on an ergometer (Xiris, Care fitness, Bobigny, France) fixed to the floor of the aircraft. Each sequence started with a 45 second phase of normogravity, preceding the parabola that was followed by the hypergravity (20 s) and microgravity (22 s) phases. The sequence ended after the microgravity phase. Rest intervals of at least 2 minutes were observed between each sequence, with an additional 5-minute break provided after sequences #5 and #10 for the participants. The bike seat was vertically aligned with the pedal hub, and the seat height was adjusted for each participant to obtain an upright cycling position when the foot pedal was down. Participants were instructed to pedal at their own beat, with the arms at their sides. They were secured with a hip harness (Samara, Petzl, Crolles, France) connected bilateraly to the floor with adjustable straps, and their feet were cliped on automatic pedals (Eggbeater, Crankbrothers, Pozzoleone, Italy). The side straps were set in normogravity to prevent the participants from leaving the seat in microgravity. So as not to interfere with pedaling, the original seat was replaced by a thin seat (Q-Bik Flow, Selle Italia, Casella d’Asolo, Italy). Two resistance settings were used on the exercises bike, one “*Easy*” setting that had almost no resistance with unbraked pedaling, and one “*Harder*” setting that had a moderate resistance (half of the maximum bike setting). Using crank integrated force transducer (Garmin, Rally 200), the required power to pedal in *Easy* and *Harder* were recorded on ground, at the average cadencies recorded in the experiment. This power was about 80 Watts in *Easy* and 200 Watts in *Harder*, at 60 rpm and 50 rpm respectively. To counteract the effects of fatigue, these resistances were pseudo-randomly distributed in the sequences for each subject.

### Pedaling kinematics

A rotative position transducer (PS-20-B-0-103, Gefran, Provaglio D’iseo, Italy) linked to an USB-6259 A/D interface (National Instrument, Austin, USA; 2KHz) was utilized to record the position of pedal-board. The pedaling rate (rpm) and angular velocity (°/s) were extracted. For the subsequent analysis, the data were divided into cycles, where a cycle was defined as a complete revolution of the right pedal, ranging from 0 to 360°, with the original point set at the bottom position. The cycles were further sorted based on gravity condition (normo-, hypo- and hypergravity) and exercise resistance (*Easy* and *Harder*).

During a pedal revolution, the angular velocity was not constant but varied throughout the movement. To analyze this variability, we identified two specific positions for each subject in each condition. These positions were as follows: (1) the lower velocity position that corresponded to the right pedal being near the top position, at 12 o’clock ; (2) the higher velocity position that was located between the front and bottom positions of the pedal, approximately at 4 o’clock. Once these positions were identified, we computed the velocity variation ratio for each subject in each condition. The velocity variation ratio represents the percentage variation between the lowest and the highest values of angular velocity observed during a pedal revolution, relative to the average velocity of the revolution. This calculation helped quantify how much the angular velocity fluctuated during the pedaling movement.

### Electromyography (EMG)

EMGs were recorded bilaterally from the biceps femoris (BF), gluteus maximus (GM), rectus femoris (RF), lateral gastrocnemius (GL) and tibialis anterior (TA). Recordings were performed using an analog amplifier (Bagnoli, Delsys, Natick, USA; x1000 amplifier) linked to the same A/D card used for kinematic data recordings (NI-USB 6259). Due to a material limitation during one flight campaign, we only obtained data from 6 subjects for the rectus femoris and biceps femoris.

Raw EMG signals from of each muscle were high-pass filtered (Butterworth filter, 30 Hz, 4^th^ order). The instantaneous frequency of EMG was computed using *instfreq* Matlab (2021b) function, and the median of the frequency (iMNF) was computed for each cycle. EMG was then rectified and a zero lag low-pass filter was applied (Butterworth filter, 3 Hz, 4^th^ order) to draw the envelope. Signals were divided into cycles based on the pedal kinematics. For each cycle, EMG amplitudes were measured between the minimal and the maximal value. To compare the EMG amplitudes across different cycles, we normalized the amplitudes for each cycle by expressing them as a percentage of the subject’s mean amplitude across all the conditions. EMG of each cycles were also normalized in the time domain and expressed from 0 to 100% of cycle duration. This step allows to align the EMG data from different cycles, making it possible to compute the average EMG across cycles for each subject based on the gravity and exercise resistance conditions.

### Muscle synergies extraction

For each condition and each muscle, EMG envelope was normalized to the average of its peak value across the cycles, as in previous studies focusing on muscle synergies^6^. Non-negative matrix factorization was performed from a set of consecutive pedaling cycles, to take into account the intercycle variability^39^. We implemented the Lee and Seung algorithm^40^ with the method described by Hug et al. ^7^. To avoid local minima, the algorithm was replicated 100 times for each subject in each condition, using “nnmf” function in Matlab. The lowest cost solution was kept (i.e., minimized squared error between original and reconstructed EMG patterns), and two synergies were extracted. The cumulative percentages of variance accounted for (VAF) explained by two synergies was > 90% in all the conditions (92%, SD = 1). For each synergy, in each condition, we extracted weighting coefficients (or muscles vectors) that specifies the relative impact of the synergy for each muscle. We also extracted the activation coefficients that specify the relative contribution of the synergies over the time (percentage of pedal revolution). based on these activation coefficients, we measured the instant in the cycle (in percentage) where the synergy contributions was maximal to the muscular activity.

### Statistical analysis

For each variable, a Kolmogorov-Smirnov test was used to ensure that the data were normally distributed. For the pedalling kinematic (cadencies and angular velocity) and EMG measures (amplitude and frequencies), repeated-measures analysis of variance were applied using Matlab “ranova” function, with Gravity (0 g, 1 g, 2 g) and resistance (*Easy*, *Harder*) as within subject factors. Post hoc analysis were performed using HSD test (“multcompare”). To discern potential behavior modifications between the initial exposure to modified gravity and later stages of the flight (such as cognitive or training bias), cadences and velocity variation ratio derived from the first 10 cycles were compared to the following cycles of the experiment (from the twentieth to the last), for each gravitational condition at each resistance level. Neurophysiological markers of fatigue were assessed by comparing the EMG amplitudes and frequencies derived from the last 10 cycles with those from the first 10 cycles for each gravitational condition at each resistance level. Repeated-measures analyses of variance were applied, considering the period of flight (beginning, ending), gravity level (0 g, 1 g, 2 g), and resistance (Easy, Harder) as within-subject factors. For synergies (weighting coefficients of each muscle, time and amplitude of activation coefficients), the main effects of Gravity (0 g, 1 g, 2 g) and Resistance (*Easy*, *Harder*) were tested used the Friedman test (“friedman”). When a main effect of gravity was present, post hoc tests were computed with Wilcoxon signed rank test (“signrank”). The level of significance was set at *P* < 0.05 in all analysis.

### Reporting summary

Further information on research design is available in the Nature Research Reporting Summary linked to this article.

### Supplementary information

Reporting Summary

## Data Availability

The data that support the findings of this study are available on the following repository: https://filesender.renater.fr/?s=download&token=d88e0ef6-c35a-474f-9a66-d7c55181ccd3.

## Code availability

The code used for the analyses presented here is available on the following repository: https://gitub.u-bordeaux.fr/etguilla/npj-microgravity-01141r.
