# Supplementary material for: Sensorimotor adaptation of locomotor synergies to gravitational constraint
Source: NPJ Microgravity. 2024 Jan 11;10:5. doi: 10.1038/s41526-024-00350-2 (PMC10784505; doi:10.1038/s41526-024-00350-2)
Supplement: Supplementary file 1 — Reporting Summary [file 41526_2024_350_MOESM1_ESM.pdf]

nature portfolio

Corresponding author(s): Etienne Guillaud

Last updated by author(s): Nov 29, 2023

Reporting Summary

Nature Portfolio wishes to improve the reproducibility of the work that we publish. This form provides structure for consistency and transparency in reporting. For further information on Nature Portfolio policies, see our Editorial Policies and the Editorial Policy Checklist.

Statistics

For all statistical analyses, confirm that the following items are present in the figure legend, table legend, main text, or Methods section.

n/a	Confirmed	
☐	☒	The exact sample size (*n*) for each experimental group/condition, given as a discrete number and unit of measurement
☐	☒	A statement on whether measurements were taken from distinct samples or whether the same sample was measured repeatedly
☐	☒	The statistical test(s) used AND whether they are one- or two-sided<br>*Only common tests should be described solely by name; describe more complex techniques in the Methods section.*
☒	☐	A description of all covariates tested
☐	☒	A description of any assumptions or corrections, such as tests of normality and adjustment for multiple comparisons
☐	☒	A full description of the statistical parameters including central tendency (e.g. means) or other basic estimates (e.g. regression coefficient) AND variation (e.g. standard deviation) or associated estimates of uncertainty (e.g. confidence intervals)
☐	☒	For null hypothesis testing, the test statistic (e.g. *F*, *t*, *r*) with confidence intervals, effect sizes, degrees of freedom and *P* value noted<br>*Give P values as exact values whenever suitable.*
☒	☐	For Bayesian analysis, information on the choice of priors and Markov chain Monte Carlo settings
☒	☐	For hierarchical and complex designs, identification of the appropriate level for tests and full reporting of outcomes
☒	☐	Estimates of effect sizes (e.g. Cohen's *d*, Pearson's *r*), indicating how they were calculated

Our web collection on statistics for biologists contains articles on many of the points above.

Software and code

Policy information about availability of computer code

Data collection: Datas were collected using Matlab R2015b and its commercial Data Acquisition toolbox recording directly the analogical input of the National Instument card (see Material and Method).

Data analysis: all analyses (EMG filter, rectification, non-negative matrix factorisation, fast-fourier transform, ...), including statistical tests, were made using Matlab R2022b and its commercial Signal Processing and Statistical Toolbox.

For manuscripts utilizing custom algorithms or software that are central to the research but not yet described in published literature, software must be made available to editors and reviewers. We strongly encourage code deposition in a community repository (e.g. GitHub). See the Nature Portfolio guidelines for submitting code & software for further information.

Data

Policy information about availability of data

All manuscripts must include a data availability statement. This statement should provide the following information, where applicable:

- Accession codes, unique identifiers, or web links for publicly available datasets
- A description of any restrictions on data availability
- For clinical datasets or third party data, please ensure that the statement adheres to our policy

The dataset is available at the following link :

https://filesender.renater.fr/?s=download&token=b7bd65b2-97c9-4c47-9ec4-897afd37ea2c
password : 01QQ%I02RnbH02L07t01O>db01cQOC02UDwk01y7ms0072QX

Research involving human participants, their data, or biological material

Policy information about studies with human participants or human data. See also policy information about sex, gender (identity/presentation), and sexual orientation and race, ethnicity and racism.

Reporting on sex and gender	The sex ratio was used and reported to characterize our sample, ensuring its representativeness of the population. No analysis was conducted as sex differences should not, a priori, influence the behaviors under study, and the division of our sample into two groups limits statistical power.
Reporting on race, ethnicity, or other socially relevant groupings	No reports on race, ethnicity, or other socially relevant groupings
Population characteristics	see above
Recruitment	The participants were all voluntary participants with no hierarchical or familial connection to the experimenters.
Ethics oversight	The procedures were approved by the French National Research Ethics Review Board (Comité de Protection des Personnes Sud-Ouest et Outre Mer III) under agreement number 2011-A00424-37

Note that full information on the approval of the study protocol must also be provided in the manuscript.

Field-specific reporting

Please select the one below that is the best fit for your research. If you are not sure, read the appropriate sections before making your selection.

☒ Life sciences ☐ Behavioural & social sciences ☐ Ecological, evolutionary & environmental sciences

For a reference copy of the document with all sections, see nature.com/documents/nr-reporting-summary-flat.pdf

Life sciences study design

All studies must disclose on these points even when the disclosure is negative.

Sample size	The sample size was constrained by access to parabolic flight, facilitated by support from the national space agency. With the opportunity for six flights and two participants per flight, our sample size comprised 12 participants with no room for expansion.
Data exclusions	No data were excluded
Replication	Because of the constrained acces to microgravity, there is no attempt at replication
Randomization	Participants were not divided into separate groups; instead, each participant underwent all experimental conditions. The presentation order of gravitational conditions was determined by the parabolic flight method.
Blinding	There is only one group, and concealing conditions was not feasible for this behavioral experiment as participants were informed about their environment through various physiological sensors.

Reporting for specific materials, systems and methods

We require information from authors about some types of materials, experimental systems and methods used in many studies. Here, indicate whether each material, system or method listed is relevant to your study. If you are not sure if a list item applies to your research, read the appropriate section before selecting a response.

Materials & experimental systems

n/a	Involved in the study	
☒	☐	Antibodies
☒	☐	Eukaryotic cell lines
☒	☐	Palaeontology and archaeology
☒	☐	Animals and other organisms
☒	☐	Clinical data
☒	☐	Dual use research of concern
☒	☐	Plants

Methods

n/a	Involved in the study	
☒	☐	ChIP-seq
☒	☐	Flow cytometry
☒	☐	MRI-based neuroimaging

Plants

Seed stocks

Report on the source of all seed stocks or other plant material used. If applicable, state the seed stock centre and catalogue number. If plant specimens were collected from the field, describe the collection location, date and sampling procedures.

Novel plant genotypes

Describe the methods by which all novel plant genotypes were produced. This includes those generated by transgenic approaches, gene editing, chemical/radiation-based mutagenesis and hybridization. For transgenic lines, describe the transformation method, the number of independent lines analyzed and the generation upon which experiments were performed. For gene-edited lines, describe the editor used, the endogenous sequence targeted for editing, the targeting guide RNA sequence (if applicable) and how the editor was applied.

Authentication

Describe any authentication procedures for each seed stock used or novel genotype generated. Describe any experiments used to assess the effect of a mutation and, where applicable, how potential secondary effects (e.g. second site T-DNA insertions, mosiacism, off-target gene editing) were examined.
